# Cardiac Fibroblast-Derived Extracellular Matrix (Biomatrix) as a Model for the Studies of Cardiac Primitive Cell Biological Properties in Normal and Pathological Adult Human Heart

**DOI:** 10.1155/2013/352370

**Published:** 2013-05-02

**Authors:** Clotilde Castaldo, Franca Di Meglio, Rita Miraglia, Anna Maria Sacco, Veronica Romano, Ciro Bancone, Alessandro Della Corte, Stefania Montagnani, Daria Nurzynska

**Affiliations:** ^1^Department of Public Health, University of Naples “Federico II”, Via S. Pansini 5, 80131 Naples, Italy; ^2^Department of Cardiothoracic Sciences, Second University of Naples, Via L. Bianchi, 80131 Naples, Italy

## Abstract

Cardiac tissue regeneration is guided by stem cells and their microenvironment. It has been recently described that both cardiac stem/primitive cells and extracellular matrix (ECM) change in pathological conditions. This study describes the method for the production of ECM typical of adult human heart in the normal and pathological conditions (ischemic heart disease) and highlights the potential use of cardiac fibroblast-derived ECM for *in vitro* studies of the interactions between ECM components and cardiac primitive cells responsible for tissue regeneration. Fibroblasts isolated from adult human normal and pathological heart with ischemic cardiomyopathy were cultured to obtain extracellular matrix (biomatrix), composed of typical extracellular matrix proteins, such as collagen and fibronectin, and matricellular proteins, laminin, and tenascin. After decellularization, this substrate was used to assess biological properties of cardiac primitive cells: proliferation and migration were stimulated by biomatrix from normal heart, while both types of biomatrix protected cardiac primitive cells from apoptosis. Our model can be used for studies of cell-matrix interactions and help to determine the biochemical cues that regulate cardiac primitive cell biological properties and guide cardiac tissue regeneration.

## 1. Introduction

Advances in stem cell biology and recent reports of stem cells and progenitors cells residing in the adult human heart gave origin to cardiovascular regenerative medicine, and this approach to the heart failure treatment immediately captured the attention of clinicians [1]. Numerous preclinical studies and clinical trials have aimed at the use of stem cells in the therapy of infarcted heart or ischemic cardiopathy [2], evolving from cell therapy to refined bioengineering. In the era of tissue engineering, there is an ongoing interest in the development of biomimetic materials that would be able to elicit specific cellular responses and direct tissue formation based on either intrinsic tissue regeneration capacity (provided by resident stem cells) or scaffold-incorporated or scaffold-seeded stem cells [3]. Several such approaches to cardiac tissue regeneration have been developed and are still under investigation (reviewed in [4]). 

All cells exist *in vivo* in a specialized environment in which their survival and function are assured, while their biological activity is controlled. Extracellular matrix (ECM), with growth factors stored within it, contributes to this microenvironment. While the expression of some ECM components, among which matricellular proteins, is typical of tissue development and organogenesis [5, 6], a number of cardiac diseases, including myocardial ischemia, are associated with qualitative and quantitative alterations in ECM proteins [7]. Cardiac resident or homing stem cells, as well as cells injected in or applied onto myocardium in cell-populated scaffolds interact with ECM components and respond to the local tissue conditions accordingly [8]. Therefore it seems reasonable that the role of ECM and, even more importantly, the effects of the modifications of its composition ongoing in pathological conditions should be studied and taken into consideration when planning the use of cardiac primitive cell-mediated tissue regeneration.

This study describes the method for the *in vitro* production of ECM typical of adult human heart in the normal and pathological conditions (ischemic heart disease) and highlights the potential use of cardiac fibroblast-derived ECM for *in vitro* studies of the interactions between ECM components and cardiac primitive cells responsible for tissue regeneration.

## 2. Materials and Methods

### 2.1. Cardiac Tissue Samples

Cardiac tissue samples were obtained from the left atrium of hearts from patients with end-stage heart failure due to ischemic heart disease, undergoing heart transplantation (*n* = 9, mean age 55.8 ± 3.1 years, 7 males, 2 females, mean ejection fraction 25 ± 1%). Samples of atrial appendages from normal hearts (*n* = 9, mean age 50.4 ± 4.1 years, 6 males, 3 females) were collected from the donor heart waste fragments, that is, tissue trimmed off from the heart while adjusting atrium size and form at the time of organ transplantation. Specimens were collected without patient identifiers following protocols approved by Monaldi Hospital and in conformity with the principles outlined in the Declaration of Helsinki.

### 2.2. Isolation of Fibroblasts and Cardiac Primitive Cells

Cardiac tissue samples were dissected, minced, and enzymatically disaggregated by incubation in 0.25% trypsin (Sigma-Aldrich, St. Louis, MO, USA) and 0.1% (w/v) collagenase II (both from Sigma-Aldrich, St. Louis, MO, USA) for 30 minutes at 37°C. The digestion was stopped by adding double volume of HBSS supplemented with 10% FBS. This preparation was further disaggregated by pipetting and tissue debris and cardiomyocytes were removed by sequential centrifugation at 100 g for 2 minutes, passage through 20 *μ*m sieve, and centrifugation at 400 g for 5 minutes.

Obtained cell population was used for isolation of fibroblasts and cardiac primitive cells by immunomagnetic cell sorting based on Miltenyi Biotec (Bergisch Gladbach, Germany) protocol. Fibroblasts were purified by positive selection with antifibroblast MicroBeads and passage through MS columns placed in magnetic field, followed by incubation of the collected negative fraction with anti-human-CD117 MicroBeads and positive selection of CD117-positive cardiac primitive cells.

### 2.3. Extracellular Matrix Deposition and Decellularization *In Vitro*

The previously published protocols [9, 10] have been modified and optimized for the culture of cardiac primitive cells from adult human heart. Cardiac fibroblasts were seeded on gelatin-coated plates in DMEM supplemented with 10% fetal bovine serum and cultured in confluent state (15 × 10^3^ cells per cm^2^) for up to 21 days, allowing for extracellular matrix (ECM) deposition. Then, fibroblasts were removed by incubation with a solution of 0.25% Triton X-100 and 10 mM NH_4_OH in PBS prewarmed to 37°C. The decellularization process was observed at an inverted phase contrast microscope (Olympus Italia, Segrate, Italy). After 1-2 minutes, when cells were no longer discernible, the decellularization solution was diluted and removed, followed by gentle washing of culture plates with PBS. Fibroblast-derived ECM was photographed with a computer-assisted digital camera connected to the microscope (Color View IIIu, Soft Imaging System, Muenster, Germany) and stored at +4°C. 

#### 2.3.1. Immunofluorescence

Both fibroblasts during ECM secretion and fibroblast-derived ECM after decellularization (biomatrix) were fixed in 4% paraformaldehyde for 20 minutes at room temperature. After blocking with 10% donkey serum, plates were incubated with primary antibody against fibronectin (rabbit polyclonal anti-human, Sigma-Aldrich), collagen IV (mouse monoclonal anti-human, Sigma-Aldrich), tenascin-C (rabbit polyclonal anti-human, Santa Cruz Biotechnology, Dallas, TX, USA), or laminin (mouse monoclonal anti-human, Sigma-Aldrich) and specific secondary antibodies conjugated with fluorescein or rhodamine (Jackson ImmunoResearch Europe, Newmarket, UK); F-actin was stained with rhodamine phalloidin (Sigma-Aldrich). Nuclei were counterstained with DAPI (Merck Millipore, Billerica, MA, USA), and the stained area of culture dish was mounted in Vectashield (Vector Labs, Burlingame, CA, USA). Microscopic analysis was performed with a Leica DMLB microscope equipped with epifluorescence EL6000 system (Leica Microsystems). Pictures were taken with digital camera connected to the microscope (Leica DFC345FX) and then merged with the software Leica Application Suite 3.6.

#### 2.3.2. Electrophoresis and Immunoblotting

Control plates were used to evaluate the number of fibroblasts when cultured in confluent state: no statistically significant difference in cell number was observed between plates with normal and pathological fibroblasts. Protein extracts were prepared from biomatrix secreted by normal (Bm-N) and pathological (Bm-P) heart-derived fibroblasts. Lysates containing 30 *μ*g of proteins were size fractionated by electrophoresis on 8% SDS-polyacrylamide gel and transferred onto a PVDF membrane (Merck Millipore). Molecular weight markers were loaded onto each gel as a weight indicator. The membranes were blocked and then incubated with one of the following antibodies: tenascin-X, laminin *α*1, laminin *α*2, fibronectin, and collagen I, followed by horseradish peroxidase-labelled secondary IgG (all from Santa Cruz Biotechnology). Antibody binding was visualized by chemiluminescence (Amersham Biosciences, Piscataway, NJ, USA) and autoradiography (Eastman Kodak Company, Rochester, NY, USA).

### 2.4. Cardiac Primitive Cell Culture

Pathological heart-derived CD117-positive cells were plated on biomatrix-covered culture dishes at a density of 8.5 × 10^3^ cells per cm^2^ in Dulbecco's modified Eagle's medium-Ham's F-12 medium (Sigma-Aldrich) supplemented with 10% fetal calf serum (Sigma-Aldrich), basic fibroblast growth factor (Peprotech, Rocky Hill, NJ, USA), glutathione (Sigma-Aldrich), penicillin, and streptomycin (Life Technologies, Paisley, UK). For evaluation of their proliferation, apoptosis, and migration, cells cultured on surface treated with bovine serum albumin (CTR), biomatrix secreted by normal (Bm-N), and pathological (Bm-P) heart-derived fibroblasts were starved in serum-free medium for 24 hours. All *in vitro* experiments were repeated a minimum of three times, in triplicate.

#### 2.4.1. Proliferation

For evaluation of proliferation, quiescent cells were incubated with the complete medium for 24 hours, and 5-bromo-2′-deoxyuridine (BrdU) was added (1 : 1.000) for 1 hour before cell fixation. Incorporation of BrdU was evaluated by immunofluorescence with the use of BrdU Labeling and Detection Kit (Roche Diagnostics, Basel, Switzerland), according to the manufacturer's protocol. Nuclei were counterstained with DAPI (Merck Millipore), and the stained area of culture dish was mounted in Vectashield (Vector Labs). Microscopic analysis was performed with a Leica DMLB microscope equipped with epifluorescence EL6000 system (Leica Microsystems).

#### 2.4.2. Apoptosis

For evaluation of apoptosis, cells were incubated with 200 *μ*M hydrogen peroxide in the complete medium for 24 hours and fixed in 1% paraformaldehyde. The fragmentation of DNA was detected using the ApopTag Plus Fluorescein In Situ Apoptosis Detection Kit (Merck Millipore) based on terminal transferase dUTP nick end labeling, according to the manufacturer's protocol. Nuclei were counterstained with DAPI (Merck Millipore), and the stained area of culture dish was mounted in Vectashield (Vector Labs). Microscopic analysis was performed with a Leica DMLB microscope equipped with epifluorescence EL6000 system (Leica Microsystems).

#### 2.4.3. Migration

For the evaluation of migration of cardiac primitive cells in the presence of biomatrix, cells were grown to confluence, and a thin scratch was introduced on culture plates with a pipette tip, producing a cell-free zone [11]. The plates were observed at an inverted phase contrast microscope (Olympus Italia) and photographed with computer-assisted digital camera (Soft Imaging System) at five distinct points, previously marked along the scratch on every culture plate, at various time points. Cell migration was quantified by measuring the width of the cell-free zone (distance between the edges of the scratched cell monolayer) at every time point with Cell^A^ Imaging Software for Life Sciences Microscopy (Soft Imaging System).

### 2.5. Statistical Analysis

All numerical data are presented as mean ± SEM. Statistical differences between groups were evaluated with Student's two-tailed unpaired *t*-test; *P* < 0.05 was considered significant.

## 3. Results and Discussion

### 3.1. Biomatrix Production and Characterization

**Figure 1:**
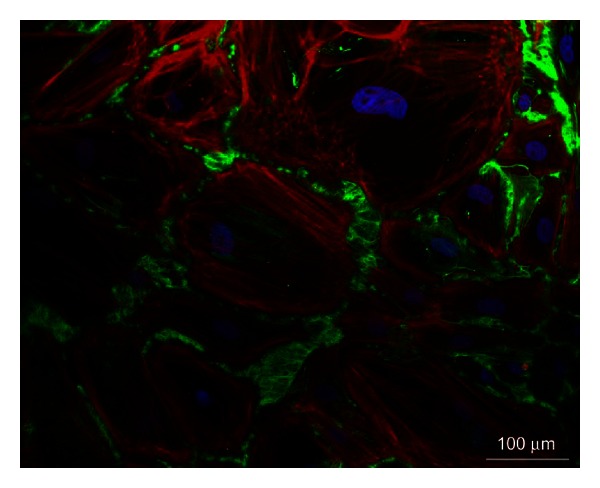
Biomatrix synthesis. Fibroblasts isolated from samples of adult human heart were cultured in confluent state allowing for ECM deposition *in vitro*. Representative image obtained by immunofluorescent labelling of actin filaments (red), cell nuclei (blue), and fibronectin (green).

**Figure 2:**
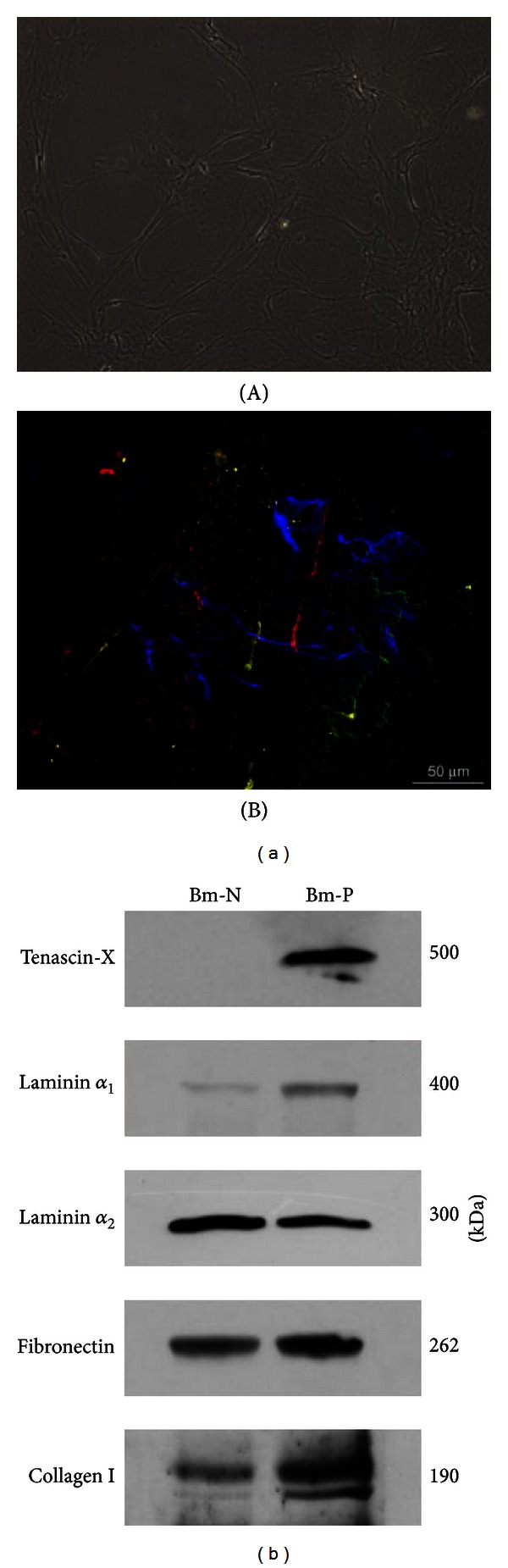
Decellularized biomatrix. (a) After nonenzymatic removal of fibroblasts, ECM was observed at phase contrast microscope (A). Its composition was revealed by indirect immunofluorescent staining of representative extracellular matrix proteins (B) collagen IV (red), laminin (green), fibronectin (blue), and tenascin-C (yellow). (b) Electrophoresis of biomatrix, followed by immunoblotting, revealed semiquantitative differences in biomatrix composition.

Fibroblasts isolated from samples of adult human heart were cultured in confluent state allowing for ECM deposition *in vitro* (Figure 1). Incubation with basic solution of Triton X-100 induced membrane permeabilization and cell lysis, resulting in the removal of fibroblasts. Decellularized matrix adhered to culture plate; its presence was observed at phase contrast microscope (Figure 2(a)), while its composition was revealed by indirect immunofluorescent staining of representative extracellular matrix proteins and glycoproteins (Figure 2(b)): fibronectin, collagen, laminin, and tenascin.

An organized ECM is necessary for the arrangement of cells and thus for the maintenance of structure in any given tissue; this intricate interlocking mesh of fibrillar and nonfibrillar proteins and glycosaminoglycans also determines tissue biomechanical properties. Numerous cell types are able to synthesise and secrete ECM components, and their activity is regulated and changes in response to various stimuli, such as inflammation, biomechanical stress, and tumorigenesis [12]. Similarly, already deposited ECM proteins are targeted by specific enzymes, metalloproteinases, that are responsible for the continuous remodeling of ECM and allow cell movement and size adaptation. In any necrotic tissue, fibronectin and collagen deposition is responsible for scar formation, which preserves wall integrity and thickness. Life-threatening pathologies ensue when ECM remodelling becomes exacerbated by chronic stimuli. Also in the ischemic heart disease or cardiac pressure overload, cardiomyocyte hypertrophy is accompanied by interstitial fibrosis, while the necrotic tissue is substituted by a scar [13]. However, in the chronic conditions such ECM remodelling increases wall stiffness, contributes to cardiomyocyte slippage, and worsens the contractile properties of the myocardium [14]. On these bases, it is becoming increasingly evident that secretory activity of fibroblasts, which exceed in number any other cell type in the myocardium, influences myocardial function: it is determinant in normal, but detrimental in pathological conditions. 

The synthetic and secretory activity of cardiac fibroblasts isolated from normal or pathological adult human heart can be studied in our model of fibroblast cell culture *in vitro*. The soluble factors condition the medium used for fibroblast maintenance, while the fibrillar and nonfibrillar proteins are deposited as biomatrix on culture plates. Both ECM components can be further analyzed by analytical methods, and their biological function can be tested on various cell types cultured in the presence of fibroblast-conditioned medium or on decellularized biomatrix. The choice of decellularization method is essential for ECM preservation [15] and subsequent use of extracellular matrix as a substrate for cell biology studies *in vitro*. Similarity in the composition and biological properties of the decellularized substrates to those of the native tissue is a prerequisite, as it grants the preserved role in the remodelling of tissue structure and modulation of cell function. In fact, many protein components of extracellular matrix react with each other and with the specific membrane receptors based on their secondary, tertiary, or quaternary structure. In our study, decellularization with nondenaturing solution allowed the preservation of protein structure. Further removal of cellular debris was accomplished by gentle washing. If molecular studies of cells cultured on naturally derived matrix are programmed, biomatrix should be treated with endonuclease before cell seeding.

### 3.2. Cardiac Primitive Cell Culture on Biomatrix

Recently, ECM proteins with a role that goes beyond the structural and mechanical support have been distinguished among other ECM components and termed matricellular proteins [16]. This group encompasses proteins that modulate cell function by interacting directly with cells or by modulating the activity of soluble factors present in extracellular microenvironment, thus influencing cell migration, proliferation, and differentiation [17]. Moreover, these proteins present unique expression pattern, with high levels during organogenesis, virtual absence in normal adult tissue, and reexpression in response to injury and tissue regeneration. In the heart, osteopontin, osteonectin, thrombospondins, tenascin, and CCN family are the matricellular proteins identified so far [18], but laminin-1 also fulfills the requirements [19]. Given these properties, extracellular matrix, and matricellular proteins in particular, can drive cardiac tissue regeneration, described in adult human heart in infarction or pressure overload [20]. Such regeneration depends on cardiac stem cells and their progenies-cardiac primitive cells committed to cardiomyocyte, endothelial, and smooth muscle cell lineage [21]. 

The biomatrix, obtained in our study by culturing fibroblasts derived from adult human heart, contained various extracellular components, including laminin and tenascin protein family. Consequently, we used this fibroblast derived-ECM as a substrate for the culture of cardiac primitive cells *in vitro*. Given the differences in biomatrix composition, revealed by western blotting (Figure 2), two types of biomatrix—Bm-N, produced by fibroblasts isolated from the fragments of adult human normal heart of donors died for reasons other than cardiovascular disease, and Bm-P, produced by fibroblasts isolated from the fragments of adult human hearts of patients with end-stage heart failure due to ischemic cardiopathy, undergoing heart transplantation—were used in this part of the study, enabling us to test the integrity and functionality of biomatrix and to compare the effects of ECM typical of normal and pathological conditions on cardiac primitive cell proliferation, apoptosis, and migration* in vitro*.

**Figure 3:**
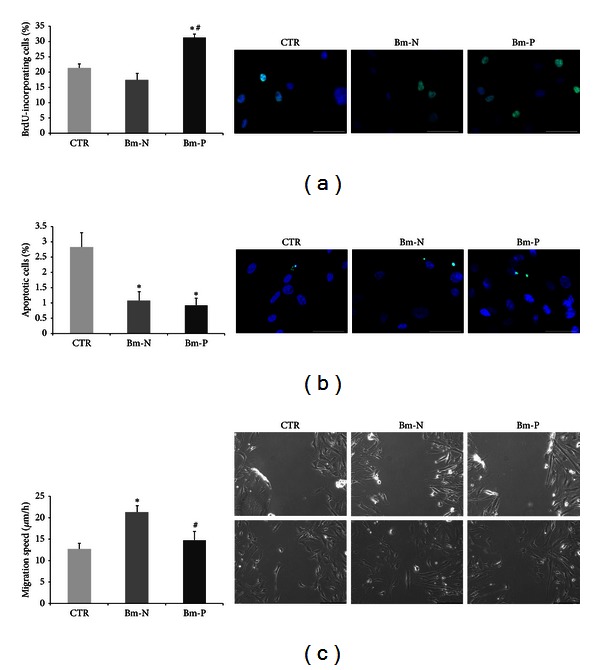
Biological characteristics of cardiac primitive cells isolated from adult human heart with ischemic heart disease cultured in the presence of ECM typical of normal and pathological adult human heart. Proliferation (a), apoptosis (b), and migration (c) were influenced by the type of the substrate. Representative images of incorporated BrdU (for evaluation of proliferation), nick end-incorporated nucleotides (for evaluation of apoptosis) immunofluorescent staining (scale bar corresponds to 50 *μ*m), and scratch wound assay at baseline and after 6 hours (for evaluation of migration, scale bar corresponds to 100 *μ*m) are shown beside the corresponding graphs. CTR: cells grown on bovine serum albumin; Bm-N: cells grown on biomatrix synthesised by fibroblasts from normal adult human hearts; Bm-P: cells grown on biomatrix synthesised by fibroblasts from hearts with ischemic cardiopathy. *P < 0,05 versus CTR; ^#^ *P* < 0,05 versus Bm-N.

Proliferation of cardiac primitive cells (Figure 3(a)) on Bm-P was 147% of control (*n* = 3, *P* < 0.01) and 177% of that in the presence of Bm-N (*n* = 3, *P* < 0.01). The presence of biomatrix protected cells from apoptosis (Figure 3(b)) provoked by oxidative stress (*n* = 6, *P* < 0.01), although no statistically significant advantage of specific biomatrix type was evident. Migration of cardiac primitive cells (Figure 3(c)) was the fastest on Bm-N; in the presence of Bm-P, it was similar to that of control but significantly slower when compared with the speed of migration on Bm-N (*n* = 9, *P* < 0.05).

It is arguable that the differences in cardiac primitive cell biological properties observed in the presence of different culture substrates (ECM deposited by normal and pathological heart-derived fibroblasts) reflect the changes in fibroblast synthesis and secretory activity, and thus in biomatrix composition. Several authors, including our group, have described the changes of cardiac stem/primitive cells biological properties in pathological conditions or aging [22, 23], but the contribution of microenvironment has not been considered and sufficiently acknowledged due to the lack of an appropriate model for cell-matrix interaction studies. The molecular constituents of ECM play major role in the responses of cells to their local microenvironment. Both direct stimulation of the specific receptors by growth factors stored in ECM and indirect activation by specific integrin expression and clustering can transmit extracellular biochemical inputs along the intracellular signaling pathways that regulate cell proliferation, survival, and migration [24]. As a matter of fact, the expression of integrin subunits in the same cardiac primitive cell population changes in qualitative and quantitative manner depending on our culture substrate (Bm-N or Bm-P, data not shown). From the above observations it follows that the composition of ECM must be taken into consideration when planning cardiac regeneration based on stem/primitive cell transplantation. Tissue regeneration can be accomplished by cells able to survive, proliferate, migrate, and differentiate in the host environment. While the methods of genetic engineering of stem cells and their application in human disease therapy are still under investigation [25], the aforementioned cell characteristics can be enhanced by cell-matrix interactions.

In mammals, nearly 300 proteins (among which collagen subunits, proteoglycans, and glycoproteins) have been identified as components of ECM [26]. So far, only few of them have been recombinantly expressed or purified and are available for the *in vitro *studies of their role in cell biology. Considering the interactions among ECM constituents, the possible influence on cardiac primitive cells is difficult to predict. Hence, our model of ECM production by cardiac fibroblasts and its use as a substrate for cardiac primitive cell culture may fill this gap and improve the results of cell transplantation. In the light of our ongoing study, the modification of biomaterials with bioactive molecules [27, 28], such as a native long chain proteins or short peptide sequences derived from intact ECM proteins, that can incur specific interactions with cell receptors should benefit from the description of biological characteristics of cells in the presence of naturally derived cardiac ECM.

In summary, this study highlights the potential use of cardiac fibroblast-derived ECM for *in vitro* studies of the interactions between components of ECM and cardiac primitive cells responsible for tissue regeneration. In the era of tissue engineering, the choice of biomimetic materials and natural biological components should be based on their specific role on cardiac cell biology. Ensuing knowledge would deepen our understanding of the biochemical cues that regulate cardiac primitive cell survival, proliferation, migration, and, possibly, differentiation, and, hence, guide cardiac tissue regeneration.
